# Meta-epigenetic shifts in T cell aging and aging-related dysfunction

**DOI:** 10.1186/s12929-025-01146-6

**Published:** 2025-05-23

**Authors:** Lorène Rousseau, Karina L. Hajdu, Ping-Chih Ho

**Affiliations:** 1 Department of Fundamental Oncology, University of Lausanne, 155 Ch. Des Boveresses, 1066 Epalinges, Switzerland; 2 Ludwig Institute for Cancer Research, University of Lausanne, Épalinges, Switzerland

**Keywords:** Immunosenescence, T cell aging, DNA methylation, Immune aging, T cell metabolism, T cell dysfunction

## Abstract

Epigenetic regulation, including DNA methylation and histone modifications, play a pivotal role in shaping T cell functionality throughout life. With aging, these epigenetic changes profoundly affect gene expression, altering T cell plasticity, activation, and differentiation. These modifications contribute significantly to immunosenescence, increasing susceptibility to infections, cancer, and autoimmune diseases. In CD8⁺ T cells, chromatin closure at key regulatory regions suppresses activation and migration, while chromatin opening in pro-inflammatory gene loci amplifies inflammation. These changes drive terminal differentiation, characterized by increased expression of senescence-associated markers, impaired migration and loss of epigenetic plasticity. CD4⁺ T cells experience fewer but critical epigenetic alterations, including disrupted pathways, a skewed Th1/Th2 balance, and reduced Treg functionality. These epigenetic changes, compounded by metabolic dysfunctions, such as mitochondrial deficiency and oxidative stress, impair T-cell adaptability and resilience in the aging organism. Therefore, understanding the interplay between epigenetic and metabolic factors in T cell aging offers promising therapeutic opportunities to mitigate immunosenescence and enhance immune function in aging populations. This review explores the interplay between DNA methylation, histone alterations, and metabolic changes underlying T cell aging.

## Introduction

### Key aspects of T cell aging

Immune cells, particularly lymphocytes, have the unique ability to persist throughout life, providing long-term immunity against disease and cancer [1]. However, aging induces significant changes in both innate and adaptive immunity, leading to a paradoxical increase in autoimmunity along with higher susceptibility to cancer and infections. While the adaptive immune system tends to become less responsive with age, chronic stimulation of the innate immune system over time results in elevated levels of circulating inflammatory mediators and increased systemic basal inflammation known as 'inflammaging' [2]. Moreover, cellular senescence directly drives inflammaging via a senescence-associated secretory phenotype (SASP) [3], characterized by the secretion of high levels of inflammatory cytokines such as IL-1b, IL-6 and IL-8. This systemic chronic inflammation underlies the pathogenesis of several age-related diseases including type-2 diabetes, rheumatoid arthritis, cardiovascular diseases, among others.

T cells are particularly affected by the aging process. One hallmark of T cell aging is a significant decline in naïve T cells, partly attributed to thymic involution—a process that begins in early adulthood. Thymic involution involves an increase in thymic adipose tissue and a loss of functional lymphoid tissue, reducing the thymus's ability to produce new naïve T cells for secondary lymphoid organs. This reduction is more pronounced in CD8+ T cells than in CD4+ T cells [4–6]. In fact, among all immune cells, the decrease in naïve CD8+ T cells is the most significant, with a reduction from 7 to 3% [7]. A potential explanation is that CD8+ T cells may undergo higher rates of homeostatic proliferation because they recognize MHC class I, which is expressed on nearly all cell types. In contrast, CD4+ T cells recognize MHC class II, which is restricted to antigen-presenting cells. Consequently, the higher frequency of stimulation might cause CD8+ T cells to reach senescence earlier than CD4+ T cells [8]. The decline in the naïve T cell population decreases TCR repertoire diversity, which may make older individuals more vulnerable to new infections. Moreover, naïve CD8+ T cells are not only fewer in number but also exhibit impaired priming capacity and an inability to mount effective de novo immune responses [9] in elderly humans. In parallel, aged memory T cells tend to become highly differentiated and dysfunctional, displaying impaired effector function, mitochondrial damage, and loss of epigenetic plasticity. This general defect in aged immune memory partly explains why older individuals often develop infections to pathogens they were previously immune to, such as the frequent varicella-zoster virus infections, or present reactivation of latent pathogens [10]. Understanding how to target age-associated T cell dysfunction can help to boost immune function in the elderly.

### T cell dysfunction in aging

In aged individuals, there is an increased frequency of late-stage differentiated memory T cells, including terminally differentiated memory cells re-expressing CD45RA (TEMRA) and effector memory T cells (Tem) [6]. These terminally differentiated memory cells downregulate costimulatory receptors CD28 and CD27 while upregulating KLRG1, which inhibits TCR-induced Akt phosphorylation and cell proliferation [11]. While these markers are commonly used to identify senescent T cells, they comprise a heterogeneous population. Some of these highly differentiated T cells exhibit true replicative senescence features, such as stable cell cycle arrest mediated by upregulation of cyclin-dependent kinase inhibitors p16 or p21, along with shortened telomeres [12]. Interestingly, senescent CD28-CD27-CD45RA+ T cells can retain effector functions, such as cytotoxic activity and cytokine secretion, despite lacking proliferative capacity [13]. Recently, it was shown that age-associated CD8+ T cells (Taa) are characterized by granzyme-K expression and elevated levels of exhaustion markers, including PD-1 and TOX, exhibiting a transcriptional profile distinct from that of younger effector-memory T cells [14]. These cells, present in both murine models and human PBMCs, closely resemble terminally exhausted cells but possess unique features, like the upregulation of tissue-homing markers. Notably, Taa CD8+ T cells could induce SASP factors expression in non-immune senescent cells, contributing to detrimental systemic inflammation. Another putative age-associated CD8+ T cell subtype are virtual memory T cells (Tvm), antigen-inexperienced naive T cells that present high levels of CD44 and mature spontaneously in response to cytokine stimulation [15]. Although these cells also occur in young individuals, they accumulate with age and show signs of DNA damage and replicative senescence even in the absence of antigen-priming [16].

**Fig. 1:**
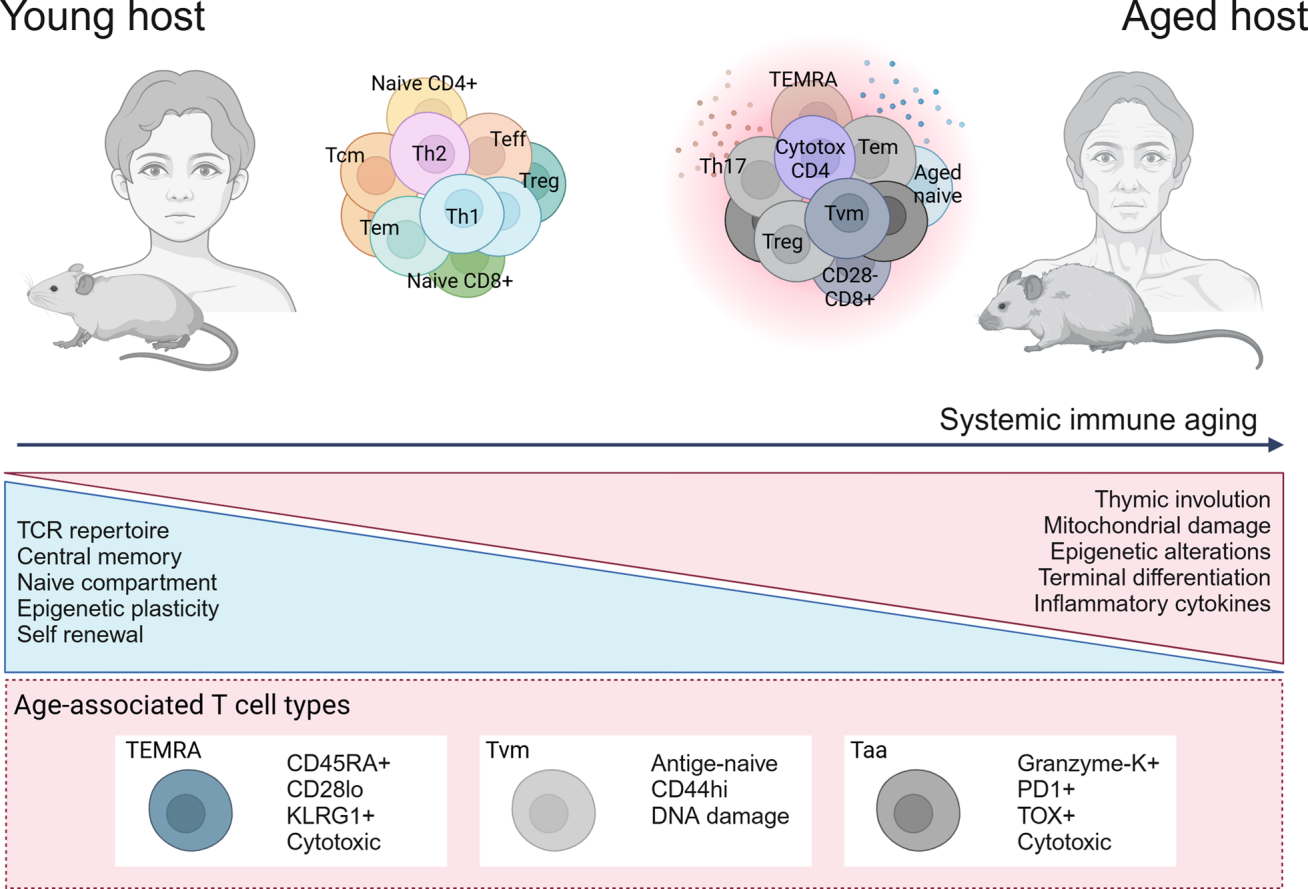
Global reorganization of T cell immunity in aging. Aging is associated with a decline in naïve T cells, driven by progressive thymic involution and reduced self-renewal capacity. Less differentiated populations, such as central memory T cells, are replaced by late-stage, highly differentiated memory phenotypes, including TEMRA and Taa cells. A hallmark of aged CD8+ T cells is the downregulation of the costimulatory receptor CD28, accompanied by upregulation of the senescence-associated marker KLRG1. Additionally, aged T cells exhibit aberrant inflammatory cytokine production. Alterations in the balance of CD4+ T cell subtypes contribute to systemic inflammation, characterized by an increased frequency of Th17 cells, a higher Th1/Th2 ratio, and impaired regulatory T cell function

CD4+ T cells also become hyperdifferentiated and dysfunctional with age, exhibiting increased activation and production of inflammatory cytokines [17]. Both effector-like CD4+ T cells and activated regulatory T cells (Tregs) increase in frequency in aged mice. CD4+ T cells from older mice show elevated PD-1 expression across several tissues, along with enhanced production of pro-inflammatory cytokines such as IL-17 and IFNγ [14]. Additionally, the number of activated Tregs increases and has been shown to directly induce CD8+ T cell senescence [18]. In contrast, another study reported that Tregs undergo accelerated senescence compared to conventional CD4+ T cells due to defective antioxidant responses, rendering them unable to protect mice from CD4+ T cell-induced colitis [19]. Mechanistically, these senescent Tregs exhibit downregulation of the E3 ligase DDB1- and CUL4-associated factor 1 (DCAF1), which interacts with GSTP1, a glutathione-S-transferase critical for ROS clearance. Current evidence suggests that although Tregs increase in number in aged individuals, their suppressive functions are impaired, contributing to systemic inflammaging. Furthermore, the frequency of cytotoxic CD4+ T cells—a typically rare population—can rise to 30% of the total CD4+ compartment in aged C57BL/6 mice [17]. This unconventional population of CD4+ T cells is also expanded among supercentenarians [20] (Fig. 1). Of note, there may be some confusion when discussing T cell aging, senescence or exhaustion. T cell senescence resembles to features of exhausted T cells observed in chronic infections and tumors, including reduced proliferative capacity, mitochondrial damage and impaired effector function [21]. However, senescence is a biological process that occurs in most cell types and refers to a state of stable replicative arrest caused by telomere shortening and accumulated DNA damage. In contrast, exhaustion is a term used specifically for T cells to describe an alternative differentiation program that typically occurs under continuous TCR stimulation, as seen in chronic infections and tumors. Importantly, not all aged T cells are senescent or truly "exhausted." Nonetheless, cellular senescence and dysfunctional T cell phenotypes contribute to the functional decline associated with T cell aging and age-related diseases.

### Epigenetic remodeling in T cell aging

T cells, like many other cell types, undergo significant genetic and epigenetic changes with aging, a phenomenon often referred to as ‘epigenetic drift’ [22]. Notably, dynamic epigenetic remodelling throughout a cell’s lifetime in response to intrinsic and environmental cues can serve as both a cause and consequence of the aging process [23]. Epigenetic regulation largely dictates T cell fates during differentiation and aging [24], and data from human samples indicates that memory CD8+ T cells exhibit the highest level of age-associated chromatin remodeling among cell types in peripheral blood mononuclear cells (PBMCs) from young and old individuals [7]. It is plausible that homeostatic proliferation of naive T cells over the course of life leads to progressive loss of naive epigenetic information, which aligns with the observed shift toward memory differentiation in aged T cells. Supporting the notion that epigenetic information is critical to sustain T cell differentiation trajectories, it was shown that T cells deficient for the histone methyltransferase DOT1L prematurely differentiate into a memory phenotype even in the absence of antigen encounter [25], a phenotype that resembles that of age-related virtual memory T cells. Moreover, Moskowitz et al. found that naive CD8+ T cells from older human donors had enhanced chromatin accessibility at genes related to differentiation compared to young naive T cells [22]. Interestingly, a recent study showed that murine memory T cells iteratively boosted with vaccination and transferred into new hosts over the course of 10 years in a multi lifetime model [26] are able to preserve telomere length and present hypermethylation in cell cycle control genes *Cdkn2a* (p19Arf, p16Ink4a) and *Cdkn2b* (p15Ink4b), with concomitant demethylation of proliferation drivers like CDK6 and Rb1 [27]. Remarkably, these cells evaded replicative senescence after 51 rounds of restimulation without showing any signs of malignant transformation. This finding suggests that cell-extrinsic factors in the host environment, such as metabolic alterations and the senescence-associated secretory phenotype, play a critical role in driving age-related immunological decline. In addition, single-cell analysis of CD4+ T cells from young and old mice following activation revealed that chronological age correlates with increased transcriptional heterogeneity among naive CD4+ cells receiving homogeneous CD3/CD28 stimulation, suggesting dysregulation in the epigenetic control of core programs for T cell function with aging [28]. Given the key role for epigenetic remodeling in age-associated T cell phenotypes and function, in this review we will discuss the current knowledge on epigenetic regulation and intersection with metabolic alterations that take place during T cell aging and age-related dysfunction.

## DNA methylation in T cell development and aging

### Introduction to DNA methylation

A key epigenetic modification involves the addition of a methyl group from S-adenosyl-l-methionine (SAM) to the 5th position of cytosine, creating a complex called 5-methylcytosine discovered by Rollin Hotchkiss in 1948 [29, 30]. DNA methylation can affect gene expression in multiple ways: First, DNA contains recognition domains for transcription factors which are usually rich in CpG motifs [31]. CpG sites are DNA regions where cytosine is followed by guanine, often subject to methylation. CpG islands are clusters of these sites, usually near gene promoters [32, 33]. When cytosine is methylated at these sites, it physically prevents transcription factors from binding to DNA by concealing their recognition sequence, as originally described by Holliday and Pugh in 1975. In addition, methylated CpG recruits specific proteins: Methyl-CpG- binding domain (MBDs) which, once bound, can inhibit gene expression [34]. In 1994, it was further revealed that these methyl groups are added by DNA methyltransferases (DNMTs) [35, 36]. DNMT1 preserves methylation patterns during cell division [37], while DNMT3a and DNMT3b are responsible for catalyzing "de novo" methylation [38]. The role of these de novo methylations is especially important in certain diseases and age-related conditions [39–43]. Recent research has focused on their critical functions in T lymphocytes. As previously mentioned, gene expression regulation plays a central role in determining the fate of lymphocytes [44]. Once activated, lymphocytes go through several stages, with the sequential expression of key genes driving their effector phase. During the contraction phase, their ultimate fate—whether they become memory cells or terminal effector cells—depends heavily on the de novo methylation added by DNMT3 enzymes [45, 46]. This was demonstrated by Brian H. Ladle, who showed that inhibiting DNMT3 activity resulted in a higher number of memory lymphocytes [47]. DNA methylation in T cell also plays a role in the long-term capacity of the cell to recall in response to re-encountering of same pathogens [48]. It has been shown that these methylation patterns evolve throughout life, and some of these chromatin rearrangements affecting key gene expression would tend to diminish the cells' ability to fight infection or cancer [49]. This intricate regulation underscores the importance of epigenetic control, particularly DNA methylation, in directing not only cellular identity but also the long-term function of immune cells.

### Alterations in aged CD8+ T Cells and CD4+ T cells

**Fig. 2:**
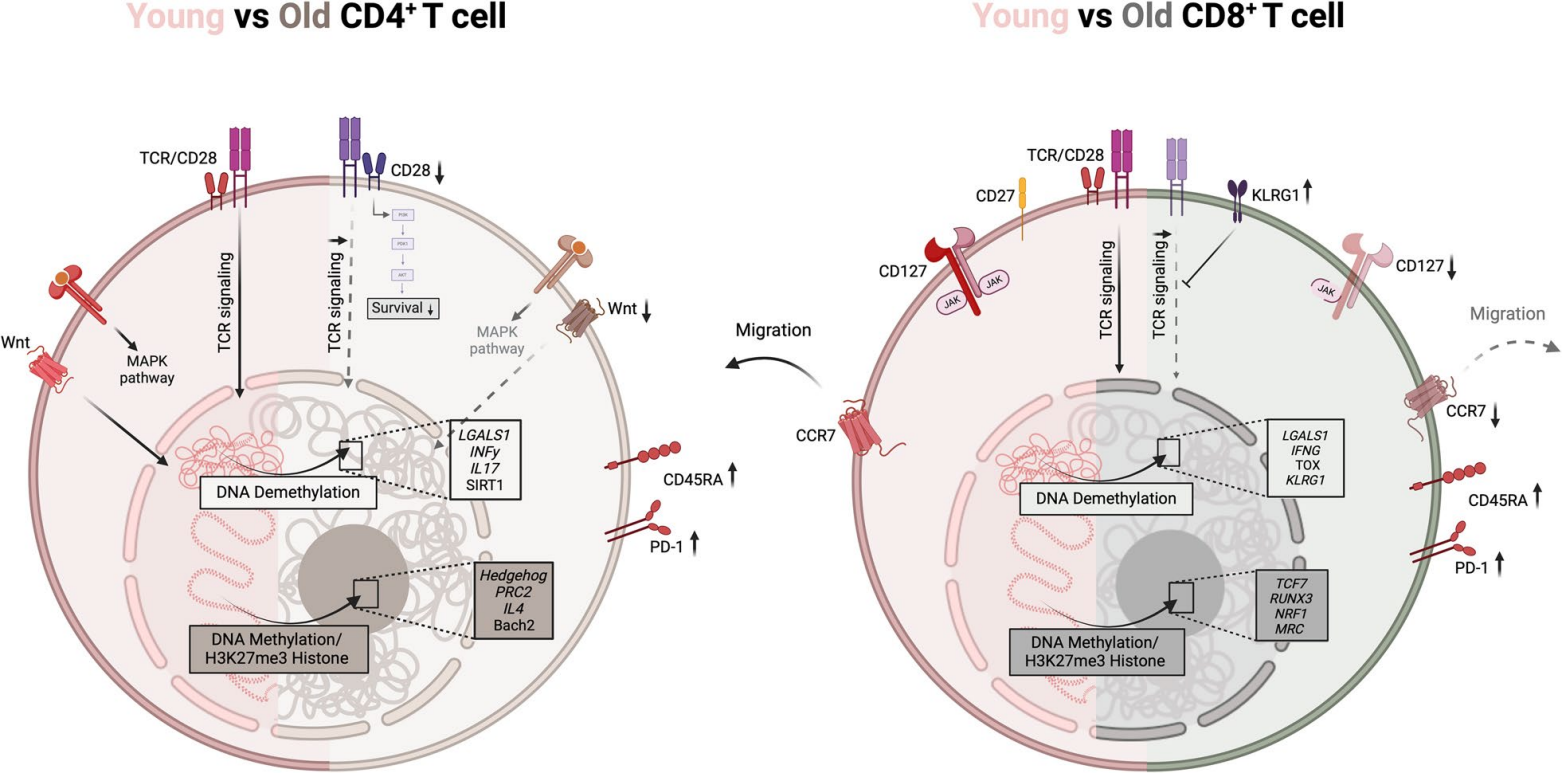
Comparison of young (pink) and aged (brown-grey) CD4⁺ and CD8⁺ T cells. Aging leads to significant epigenetic modifications in CD4⁺ and CD8⁺ T cells, altering their functionality and promoting immunosenescence. Increased DNA methylation and repressive histone marks (H3K27me3) silence key genes involved in cell plasticity, activation, migration and differentiation. At the same time, hypomethylation of pro-inflammatory genes amplifies cytokine production and inflammation, together reducing global epigenetic plasticity. These modifications alter key signaling pathways and transcriptional programs. TCR signaling is disrupted by repression of co-stimulatory molecules such as CD28 and CD27, and upregulation of KLRG1. Cells show a sharp decrease in naive populations and evolution towards terminally differentiated effector cells, re-expressing CD45RA and overexpressing PD-1. In CD8 cells, the reduction of CCR7 and CD127 compromises migration and homeostasis. CD4⁺ T cells undergo fewer but still significant epigenetic modifications, disrupting specifically the Wnt, Hedgehog and MAPK pathways. As well as a Th1/Th2 imbalance favored by IFNG hypomethylation and IL4 hypermethylation. Together these alterations increase susceptibility to autoimmune diseases and favor a terminally differential phenotype

T lymphocytes undergo significant developmental phases to ensure rapid and effective responses against pathogens. Naive T cells must remain in a state of readiness, maintaining plasticity and viability until they encounter specific antigens based on T cell receptor-peptide loaded MHC interaction [1, 50]. Upon activation, they proliferate extensively, generating an army of effector cells that target and neutralize pathogens [50, 51]. Following this expansion phase, a contraction phase ensues to downscale the immune responses, preserving only a subset of long-lived memory cells poised to respond upon re-exposure to the same antigen [45, 51–55]. This adaptability is partly driven by epigenetic regulations, notably DNA methylation, which modulate gene expression according to the requirements of each stage. Thus, methylation helps maintain the plasticity of naive T cells, promotes their proliferation in response to antigen activation, and contributes to their differentiation into effector or memory cells [37, 46, 47]. With aging, however, these epigenetic modifications evolve, leading to metabolic and functional changes in T cells. This shift reflects a broader decline in the immune responses, with aged T cells demonstrating altered metabolic profiles [48, 49, 56]. Methylation changes are mainly confined to bivalent chromatin regions [57]. These regions, located close to the promoters of poorly expressed genes, are DNA regulatory zones that allow simultaneous interaction between transcriptional repressors and activators. They are essential for maintaining cellular plasticity and avoiding early expression of genes involved in differentiation [58, 59]. Hypermethylation marks were also found in regions containing enhancers that are involved in T cell differentiation and major signaling pathways [7] as well as regions containing specific genes involved in regulatory tracts that enable the production of the different T cell subsets [22]. On the other hand, epigenetic alterations will also operate in the inverse effect, i.e. by demethylating some DNA regions that will consequently be more expressed, the regions affected would be mainly regions that are poorly expressed in young lymphocytes. Furthermore, it has been observed that these areas, are highly variable from one individual to the other, compared with hypermethylation, where the localization is more consistent between individuals [7, 60] raising the question of the heterogeneity of immune responses, particularly with ageing. In the context of aging, epigenetic alterations with DNA closing on areas more often expressed (promoters and enhancers) and DNA opening on areas normally repressed could contribute to a progressive decline in the effectiveness and diversity of the immune response (Fig. 2).

### CD8+ T cells and age-related methylation changes

Studies, including research by Liina Tserel [61] and Duygu Ucar [7] have focused on the impact of epigenetics in aged CD8+ T cells. They have revealed that an accumulation of DNA methylation in lymphocytes significantly contributes to their age-related functional decline. As previously mentioned, this phenomenon is particularly marked in CD8+ T cells when compared to CD4+ T cells. The decline in CD8+ T cell function associated with aging is linked to hypermethylation of critical genes involved in immune responses and T cell differentiation, which reduces their ability to adequately meet immunological demands. Therefore, the CD8+ T cell population undergoes a progressive evolution, marked by a significant decrease in the total number of naive CD8+ T cells, a progressive shift towards terminally differentiated effector cells, and dysfunctional memory cells characterized by reduced reactivity to antigens. These studies found a direct link between DNA methylation and the downregulation of 272 specific immune-related genes in aged CD8+ T cells from PBMCs of healthy donors [61]. For example, CD127, which encodes the IL-7 receptor, was one of the main regions identified as highly methylated and therefore very lowly expressed in aged T cells. Additionally, genes involved in the IL-7 signaling pathway, such as those encoding JAK-STAT also displayed reduced expression. The IL-7 signaling pathway plays an essential role in CD8+ T lymphocytes, as it is involved in both their development and in the normal immune response. Alteration of this pathway may result in a significant impact on CD8+ T cell function, particularly in maintaining their efficacy in the aged immune system. Other genes, such as *CCR7* and *CD27*, which are essential for T cell activation and migration [62, 63], were both hypermethylated, resulting in reduced expression in aged CD8+ T cells [61]. Similarly, genes like SATB1, TCF7, BCL11B, and RUNX3, which are essential transcriptional regulators for CD8+ T cell differentiation, are hypermethylated in aged CD8+ T cells, leading to decreased expression and impaired function [61]. SATB1 organizes chromatin to control expression of specific genes, but the deficiency of SATB1 leads to the generation of an inappropriate T cell state [64]. BCL11B, which regulates cell maturation and CD8+ T differentiation programs, plays a role in DNA damage repair during the expansion phase [65].TCF7 supports the development of memory T cells, the maintenance of naïve T cells, and the stem-cell like phenotype during chronic infection [66, 67]. RUNX3 plays an important role in migration and differentiation in memory CD8+ T cell [68]. Interestingly, the regions of DNA that are hypermethylated are mainly located in CpG island of gene regulatory region and in the region where histone repressor marks (H3K27me3 and H3K9me3) are present [61]. In contrast, the *LGALS1* gene, which codes for the immunosuppressive protein galectin-1, leading to apoptosis of effector CD8+ T cells, was observed to be demethylated and consequently overexpressed in aged CD8+ T cells. Additionally, pro-inflammatory cytokine genes such as *IFNG* and *CCL5* exhibited hypomethylation, leading to increased expression levels in these older CD8+ T cells [61]. Overall, the study by Moskowitz [22] and Ucar [7], found that these epigenetic changes mainly affected naïve and memory CD8+ T cells. In the naïve CD8+ T cells, enhancer-rich regions were particularly affected by increased chromatin closure, while in the memory cells, it was more likely to occur in regions with promoters [7, 22]. In conclusion, this significant shift in transcriptional activity likely plays a key role in the decline of immune function seen in aging individuals. Overall, these findings suggest that age-related DNA methylation changes in CD8+ T cells might drive immunosenescence by modulating the expression of crucial genes involved in immune responses and in cell differentiation.

### CD4+ T cells and age-related methylation changes

In contrast to CD8+ T cells, CD4+ T cells experience fewer methylation changes with age, and only 20 specific immune-related genes in aged CD4+ T were found [61], which is consistent with the fact that the CD4+ pool is better maintained with age [69]. Furthermore, the specific IL7R profile observed in aged CD8+ T cells was not found in aged CD4+ T cells [7]. This distinction highlights an important divergence between these two T cell subpopulations, reflecting their functional diversity and different responses to the aging processes. However, some of these modifications have been associated with altered immune functions. The LGALS1 gene, as in CD8+ T cells, was similarly overexpressed in aged CD4+ T cells [61]. Age-related methylation changes in CD4+ T cells have distinct consequences depending on whether a gene undergoes methylation or demethylation [70]. Genes that become demethylated with aging are often involved in regulating the expression of specific genes, while regions that become hypermethylated predominantly correspond to protein-coding genes. Notably, hypermethylated regions include components of the Hedgehog signaling pathway, which is crucial for cell differentiation, particularly in embryogenesis [71]. Among these, the Enhancer of Zeste Homolog 2 (EZH2), an enzyme responsible for histone-lysine N-methyltransferase activity, plays a role in histone methylation and subsequent DNA repression. EZH2 functions as part of the Polycomb Repressive Complex 2 (PRC2), which also includes SUZ12, a protein involved in chromatin silencing [72]. In addition, other pathways such as Wnt and MAPK, which are implicated in autoimmune diseases [73, 74], also exhibit increased methylation in aged CD4+ T cells [70]. These epigenetic modifications may contribute to altered signaling and diminished immune functionality in aging individuals. In addition, aging is known to shift the Th1/Th2 balance [75], leading to a predominance of Th1 responses, linked to a higher risk of autoimmune diseases [76]. A study using murine models has demonstrated that the promoter region of IFN-γ is hypermethylated in young individuals, while the promoter of IL-4 is demethylated, resulting in higher IL-4 levels and a Th2-biased immune response [77]. However, this pattern is reversed in aged mice, characterized by that IFN-γ promoters become hypomethylated and IL-4 promoters are hypermethylated, driving a Th1-skewed responses [77]. This age-associated epigenetic reprogramming disrupts the delicate Th1/Th2 balance, amplifying Th1-driven inflammation and contributing to the onset of autoimmune diseases in older individuals. In summary, age-related epigenetic changes in CD4+ T cells, while less extensive than in CD8+ T cells, have distinct impacts on immune functionality, including alterations in key signaling pathways and the Th1/Th2 balance, contributing to increased inflammation and susceptibility to autoimmune diseases in aging individuals. Understanding these mechanisms underscores the central role of DNA methylation in immune aging and highlights potential pathways to restore immune competence in the elderly.

## Histone post-translational modifications in T cell aging

The primary level of chromatin organization is the nucleosome, a structure where DNA wraps around an octamer of core histones (H2A, H2B, H3, and H4). Histones are highly conserved proteins with positively charged N-terminal tails rich in lysine and arginine residues, enabling them to interact with neighbouring nucleosomes. Post-translational modifications (PTMs) of histones play a critical role in regulating chromatin structure and gene expression [78]. These modifications are mediated by a variety of enzymes that add, recognize, or remove such marks. PTMs include acetylation, phosphorylation, methylation, ubiquitylation, and SUMOylation, among others recently described [79]. The most extensively studied PTMs are histone acetylation and methylation, for which the main regulatory proteins are well characterized. Histone acetylation on lysine residues is enriched at active promoters and enhancers, facilitated by histone acetyltransferases (HATs), and reversed by histone deacetylases (HDACs). Generally, acetylation reduces the positive charge of histones, weakening DNA-histone interactions and making DNA more accessible to the transcriptional machinery [80]. Methylation, on the other hand, can occur on all three basic amino acids: lysine, arginine, and histidine. The functional outcome of methylation depends on the specific residue modified and the degree of methylation (mono-, di-, or trimethylation). For instance, trimethylation of lysine residues like H3K9 and H3K27 is typically associated with repressive chromatin states, whereas H3K4me3 at transcription start sites is linked to active transcription [81].

Early research on yeast aging established that histone expression and post-translational modifications are involved in cellular aging [82, 83]. Given the complexity and diversity of histone modifications and their associated proteins, relatively few studies have investigated how these marks change during T cell aging. Most current insights come from studies linking DNA methylation to the concurrent presence of repressive histone marks or from research on whole-genome chromatin accessibility. Dozmorov et al. analyzed DNA methylation profiles and ChIP-seq data from naïve CD4+ T cells of human subjects aged 19 to 66 years. This study revealed that age-associated CpG sites are predominantly hypermethylated and exhibit enrichment of H3K27me3, a modification associated with PRC2 activity [83]. Consistent with studies in other cell types showing a global reduction of histones with aging, recent findings revealed that activated CD4+ T cells from older individuals exhibit reduced transcript levels of histone genes. This reduction was linked to slower S-phase progression and replicative stress. The decrease in histones was attributed to increased HDAC activity mediated by SIRT1, which bound to histone gene promoters and reduced H3K9/14Ac acetylation levels. Notably, replication stress in aged CD4+ T cells triggered the expression of inflammatory mediators, which could be reversed by SIRT1 inhibition [84]. These findings are in line with a previous report that detected significantly less chromatin accessibility in histone genes in T cells from PBMCs of older individuals [7]. Importantly, this study also showed that aging correlates with increased chromatin accessibility in previously repressed regions, supporting the ‘loss of heterochromatin’ theory of aging [85]. This theory proposes that aging gradually erodes heterochromatin regions established early in development, leading to aberrant gene expression and genomic instability [85].

Studies establishing causal links between age-related factors, specific chromatin alterations, and T cell aging remain scarce. One study demonstrated that menin deficiency in CD4+ T cells leads to reduced expression of Bach2, a critical factor for maintaining T cell homeostasis and stemness [86]. This deficiency accelerated cellular senescence and increased pro-inflammatory cytokine production. Menin was found to bind directly to the Bach2 locus, promoting histone acetylation and sustaining Bach2 expression. In contrast, menin-depleted T cells exhibited elevated H3K27me3 levels and a marked reduction in H3K27ac. Treatment with an HDAC inhibitor restored Bach2 levels by reversing these histone changes. The authors proposed that menin forms a previously unrecognized complex with the HAT PCAF to promote acetylation at the Bach2 locus [87]. Whether this same mechanism applies to CD8+ T cells warrants further investigation. Importantly, since many histone-modifying enzymes are metabolically responsive and rely on metabolites such as acetyl-CoA for acetylation and S-adenosylmethionine for methylation, there is a clear connection between cellular metabolic states and epigenetic regulation. How age-related metabolic alterations impact epigenetic remodeling, and vice versa, remains an area of active investigation.

## Metabolic- epigenetic crosstalk in T cell aging

Aging is accompanied by drastic changes in systemic and cellular metabolism. In fact, for a long time the ‘Free radical theory of aging’ [88] suggested that defects in mitochondrial function due to free radicals’ exposure over time were largely responsible for cellular senescence. Although this notion has been replaced with a more holistic view of aging as a multifactorial process, the implication of metabolic alterations and concurrent epigenetic remodeling is a key aspect of aging.

**Fig. 3:**
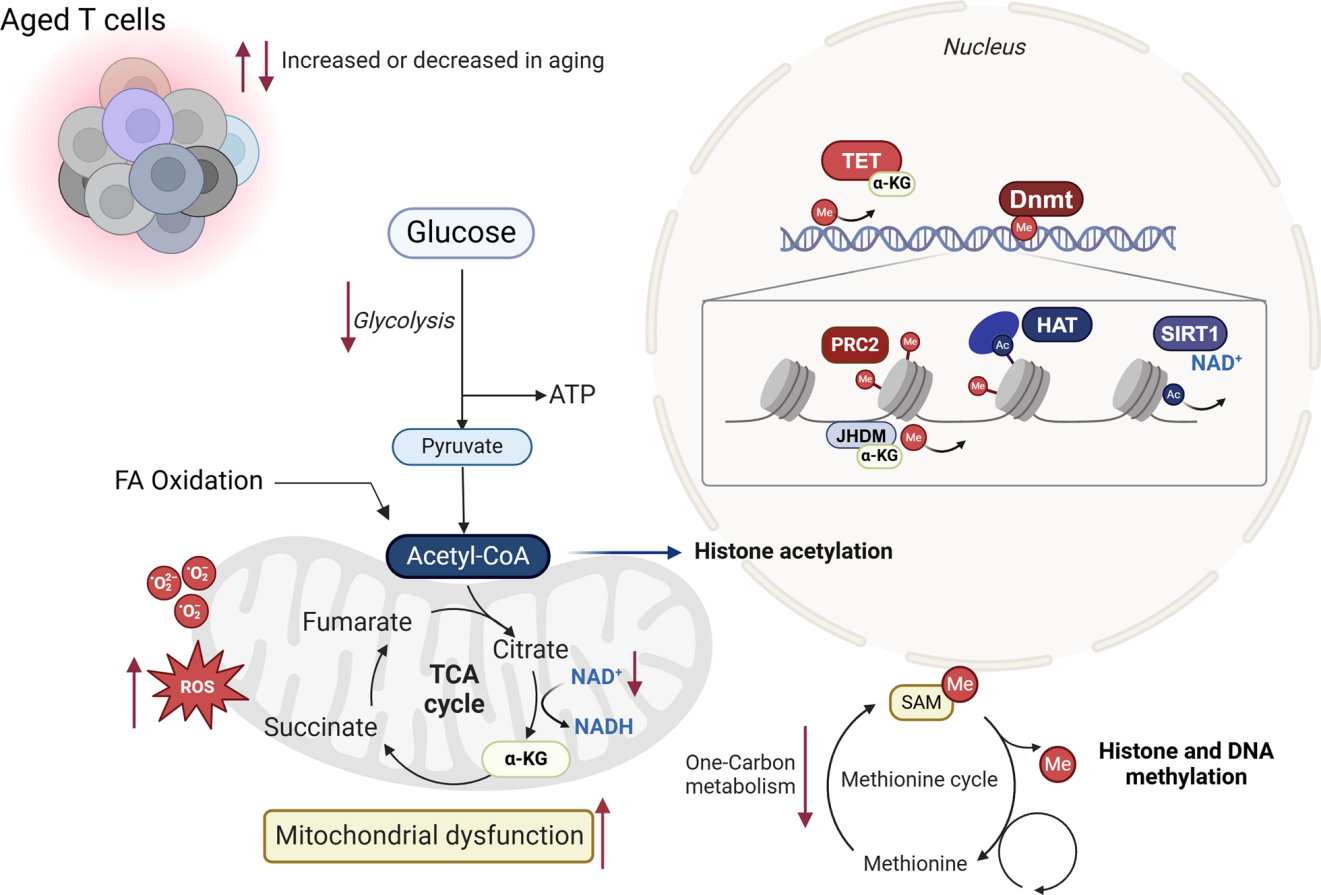
Metabolic-epigenetic crosstalk in aged T cells. Metabolic alterations and epigenetic remodeling are tightly interconnected, with many metabolites involved in epigenetic regulation originating as byproducts of mitochondrial metabolism. Mitochondrial dysfunction is a hallmark of T cell aging, leading to impaired production of several metabolites essential for epigenetic regulation. For example, α-ketoglutarate (αKG), a critical cofactor for epigenetic enzymes such as TET (DNA demethylases) and histone demethylases of the JmjC-domain-containing (JHDM) family, is reduced in aged T cells. Similarly, NAD+, required for the activity of histone deacetylases (HDACs) in the sirtuin family, such as SIRT1, is also diminished. Aged T cells exhibit defects in one-carbon metabolism, compromising the production of S-adenosyl methionine (SAM), the universal methyl donor for DNA and histone methylation. Furthermore, aged T cells accumulate reactive oxygen species (ROS), which exacerbate mitochondrial damage and directly inhibit TET activity. FA-Fatty acid, ROS-reactive oxygen species, TCA- tricarboxylic acid cycle, a-KG- a-ketoglutarate, TET- ten-eleven translocase, PRC2- Polycomb Repressive Complex 2, HAT- histone acetyl transferase, SAM- S-adenosyl methionine, JHDM- JmjC-domain-containing histone demethylation protein

Metabolic alterations and epigenetic remodeling are highly interdependent processes. Several metabolites involved in epigenetic regulation are byproducts of mitochondrial metabolism. For example, acetyl-CoA, the primary substrate for histone acetylation, is generated through the catabolism of acetate, citrate, or pyruvate, as well as via fatty acid oxidation. Additionally, the TCA cycle produces metabolites that serve as cofactors for epigenetic-modulating enzymes [89]. Histone demethylases of the JmjC-domain-containing (JHDM) family and TET DNA demethylases both require α-ketoglutarate (α-KG) as a cofactor [90, 91], Similarly, sirtuin family HDACs depend on NAD+ [92], which is synthesized either de novo via the kynurenine pathway or as a byproduct of the electron transport chain. Furthermore, SAM, synthesized via one-carbon (1C) metabolism, is the primary donor of methyl groups used by histone methyltransferases (HMTs) (Fig. 3). Interestingly, metabolomic analysis of activated naïve CD4+ T cells from aged mice revealed lower levels of several metabolites compared to younger cells, particularly those involved in the 1C pathway. As one-carbon metabolism is a critical pathway for T cell activation, the authors showed that aged CD4 T cell function could be partially restored by providing formate and glycine to the aged cells [93]. This metabolic defect also suggests that aged T cells may have reduced SAM levels for DNA and histone methylation, though this was not directly tested in the study.

### Mitochondrial dysfunction

During activation, T cells rely on rapid energy production through glycolysis, supported by the pentose phosphate pathway and glutamine metabolism for biosynthesis and other essential functions [94–96] As T cells transition to their memory phase, their metabolic focus shifts back to oxidative phosphorylation (OXPHOS) and fatty acid oxidation, ensuring efficient energy use and long-term survival [97]. Memory T cells are particularly reliant on OXPHOS, which is supported by lipid metabolism to sustain their survival [45]. Their spare respiratory capacity (SRC) enables rapid reactivation upon reinfection, underscoring the importance of mitochondrial health and metabolic adaptability [98]. The ability to switch between metabolic states, such as glycolysis during activation and OXPHOS during memory maintenance, is crucial for T cells to meet their diverse functional demands. Understanding how these metabolic switches behave in aging T cells is key to uncovering the reasons behind their functional decline and may offer insights into therapeutic strategies to restore immune resilience and improve health during aging.

Previous studies have shown that mitochondrial dysfunction is a common trait of aging in multiple cell types, including T cells. In fact, mitochondrial damage and disturbed dynamics are one of the common traits between aged and exhausted T cells [93, 99, 100]. Mitochondrial dysfunction in aging T cells is marked by reduced respiratory activity and a decline in turnover efficiency. A critical factor influencing mitochondrial mass and metabolic flexibility is CD28 co-stimulation, which supports the maintenance of mitochondrial health and metabolic adaptability. CD4+ T cells retain CD28 expression longer during aging compared to CD8+ T cells, which lose this co-stimulatory signal earlier [69, 101]. This loss in CD8+ T cells is associated with reduced mitochondrial mass and impaired metabolic switching, contributing to their greater susceptibility to functional decline [102]. Paradoxically, while aged CD8+ T cells present a decreased oxygen consumption rate (OCR), this is accompanied by an increase in mitochondrial mass, likely a compensatory mechanism that ultimately fails to restore functional energy production. A recent study showed that mice with T cells deficient for the mitochondrial transcription factor A (TFAM), an essential transcription factor for mitochondrial DNA replication, present global accelerated aging and premature death [93], highlighting the importance of mitochondrial homeostasis for normal T cell development and function. Moreover, CD4+ T cells deficient for TFAM displayed a highly differentiated Th1 phenotype, exacerbating systemic inflammation, in line with previous studies on aged CD4+ cells.

Recent findings highlight the pivotal role of Nuclear respiratory factor 1 (NRF1) in maintaining metabolic and epigenetic balance in young naïve CD8+ T cells [103]. NRF1 binding activity is essential for preserving open chromatin at key genomic regions linked to mitochondrial and metabolic gene expression. ChIP-seq data showed that NRF1 is closely associated with actively expressed regions of DNA in young naïve CD8+ T cells, where it regulates the transcription of genes critical for mitochondrial function, particularly those of the mitochondrial respiratory chain complex (MRC). On the other hand, aged naive CD8+ T cells present loss of promoter accessibility in NRF1-binding chromatin sites, including those regulating respiratory chain genes, which may explain the decline in mitochondrial biogenesis with age. In addition to electron transport chain complex genes, NRF1 controls transcription of TFAM and other critical genes involved in mitochondrial homeostasis and oxidative stress control. A follow-up study by the same group further showed that CD4+ T cells from older adults can better sustain accessibility in NRF1 binding motifs compared to CD8 T cells, indicating one potential mechanism involved in CD4 T cell resilience to aging [104]. Supporting this finding, senescent CD4+ TEMRA cells were found to have higher mitochondrial mass than their CD8+ TEMRA counterparts, along with increased PGC1a expression and enhanced oxidative metabolism [102]. However, CD4+ T cells from older human donors also showed impaired mitophagy, leading to the accumulation of dysfunctional mitochondria—a defect likely shared with CD8+ T cells [105].

### Oxidative stress

The accumulation of reactive oxygen species (ROS) in aged T cells exacerbates mitochondrial damage and further impairs cellular function. The increased oxidative stress overwhelms the cell's antioxidant defenses, leading to oxidative damage of proteins, lipids, and DNA, which accelerates cellular aging and senescence. Furthermore, reactive oxygen species (ROS) and hypoxic conditions can influence DNA demethylation mediated by TET enzymes [106]. Both CD4+ and CD8+ T cells are subject to oxidative stress as they age. The accumulation of reactive oxygen species (ROS) damages cellular components, including mitochondria. However, due to their higher mitochondrial mass, CD4+ T cells are believed to be better equipped to buffer the damaging effects of ROS compared to CD8+ T cells. This resilience allows CD4+ T cells to maintain their metabolic flexibility and adapt to the energy demands of proliferation and migration upon antigen encounter. This adaptability supports their superior capacity to respond to immune challenges, even in the context of aging.

As discussed before, aging is associated with the hypermethylation of many CpG sites in T cells, leading to decreased functionality by suppressing the expression of critical genes. One study highlighted the gene glutathione S-transferase mu 1 (GSTM1), which plays a key role in reducing oxidative stress [107]. GSTM1 is also linked to age-related conditions such as macular degeneration [108], but in human lymphocytes, its hypermethylation begins around the age of 50. This epigenetic alteration reduces GSTM1's function, increasing oxidative stress and impairing T cell functionality. In CD4+ T cells, these changes affect their differentiation into Th1 cells, which are critical for intracellular pathogen defense. Hyper-methylation suppresses the expression of T-bet, a vital transcription factor for Th1 cell development and function. As a result, CD4+ T cells in aged individuals exhibit a reduced capacity for effective immune responses, particularly against intracellular pathogens. This decline in Th1 differentiation may contribute to the immune system's diminished ability to combat infections and maintain balance during aging. While the Th1/Th2 ratio may increase with aging [75] due to a relative decrease in Th2 cells, the absolute population of both Th1 and Th2 cells declines, which ultimately weakens overall immune function and may exacerbate autoimmune tendencies associated with aging. This decline in Th1 differentiation may contribute to the immune system's diminished ability to combat infections and maintain balance during aging. Finally, it was also shown that aged memory CD8+ T cells present a decrease in chromatin accessibility in the region encoding Hypoxia-inducible factor 1-alpha (HIF1A) [7], a key regulator of the hypoxic response [109]. HIF1A plays a crucial role in cellular adaptation to low oxygen conditions by regulating genes involved in metabolism, angiogenesis, and survival. The epigenetic silencing of HIF1A in aged memory CD8+ T cells has been speculated to impair their ability to respond to hypoxic stress, further compromising their function and survival.

## T cell inflammaging in aging-related diseases

As discussed above, aged T cells undergo meta-epigenetic modifications that impair their function while contributing to a persistent, low-grade inflammatory state known as inflammaging, which plays a critical role in the onset and progression of numerous age-related diseases. Among these alterations, CD8+ T cells that lack CD28 and express senescence markers such as KLRG1 produce SASP factors like IL-6 and TNF-α [15]. These inflammatory signals not only contribute to systemic inflammation but also reinforce immune cell aging. For example, individuals with type 2 diabetes or in pre-diabetic stage exhibit elevated levels of SASP-producing senescent CD4+ and CD8+ T cells, which amplify the pro-inflammatory environment characteristic of the disease [110]. In atherosclerosis, aged CD8+ T cells accumulate within arterial plaques, releasing cytokines that promote lesion instability [111]. Similarly, in neurodegenerative disorders such as Alzheimer’s disease or Parkinson, dysfunctional CD4+ T cells infiltrate the central nervous system and contribute to chronic neuroinflammation [112, 113]. There is a bidirectional relationship between chronic inflammation and epigenetic dysregulation. For instance, age-associated loss of heterochromatin in specific genomic regions can increase the immunogenicity of nuclear DNA, thereby enhancing inflammatory signaling [114]. Conversely, pro-inflammatory cytokines such as IL-6 promote hypomethylation of repetitive DNA elements, while persistent DNA damage over time may disrupt the localization of DNA methylation enzymes, leading to further chromatin disorganization [115]. This self-sustaining cycle drives and amplifies T cell senescence [82], fueling inflammaging and contributing to the pathogenesis of numerous diseases.

## Conclusion

T-cell aging is a complex process that impacts immunity at the cellular, molecular and systemic levels. Aging disrupts the balance between activation, regulation and maintenance of the immune system, affecting the functional diversity and adaptability of CD8+ and CD4+ T cells. Age-related epigenetic alterations such as DNA methylation and histone modifications play a key role in remodeling T cell functionality, leading to altered responses to pathogens and increased susceptibility to autoimmune diseases. Moreover, the accumulation of dysfunctional, pro-inflammatory T cells contributes to inflammaging, that plays a central role in the development of multiple age-related diseases, including cardiovascular, neurodegenerative, and metabolic disorders. These changes are closely linked to metabolic reprogramming, where mitochondrial dysfunction and oxidative stress further exacerbate immune decline. While the cause and consequence relationships between metabolic alterations, epigenetic changes and T cell dysfunction in aging are still largely unexplored, this complex interplay emerges as a key factor in immunosenescence, revealing potential new therapeutic avenues to be explored. Interventions targeting metabolic pathways, epigenetic modulators, or both, could restore aged T-cell function, offering the hope of improving immunity in aging populations.

## Data Availability

Not relevant for this review study.
